# Supplementary material for: Examining the alignment between subjective effort and objective force production
Source: PLoS One. 2024 Aug 9;19(8):e0307994. doi: 10.1371/journal.pone.0307994 (PMC11315346; doi:10.1371/journal.pone.0307994)
Supplement: Attachment — Submitted filename: 2024_Rewitz_PlosOne_SV_E_objective_force_review.docx [file pone.0307994.s001.docx]

The manuscript has two main focuses. One is to study the mismatch between objective and subjective force exertion and how this mismatch can be altered by fatigue/time-on-task. The second focus is to build upon the idea that physical effort depends on the expected value of control (EVC) model developed by Shenhav, Botvinick and Cohen in 2013, as has already been suggested in previous publications, and to test whether effort is allocated differently when one anticipates to exert only physical effort versus when one anticipates to exert both physical and mental effort, with the idea that both could share a limited and quickly depletable resource. The three specific claims are that 1) subjective ratings of perceived exertion (RPE) are not matching objective force intensity linearly in a handgrip squeezing task, especially for high (60-80%) force levels, 2) time induced a reduction in the objective force exerted over time for constant RPE levels, and 3) that there is an interaction between time-on-task, and the anticipation of physical effort alone vs the anticipation of physical and mental effort.

Overall, I found the article well-written and easy to follow and read. I think that some of the results are quite interesting, either because they replicate some results (claims 1 and 2) with some new results specific to their study (claim 2) or because they discovered something new that could reflect interesting processes about how the brain proceeds with effort investment (claim 3). However, I think that the current version has some major flows, especially regarding the methodological description and the way the results are presented, and I believe that a major revision could help to significantly improve the quality of the paper.

Major comments

- The authors seem to put the three claims on the same scale, but a vast literature already exists for claim 1 that the authors seem to ignore or to set aside. I think that the manuscript should not put the three claims on the same level to highlight what is in the order of replication and what is really new in their study.
- Concerning claim 1:
  - the authors present the fact that there is a non-linear relationship between objective and subjective force exertion as something new, or at least they omit to mention that this is widely known for decades. However, this result was already presented in the initial work from Gunnar Borg, that the authors cite already in the manuscript, in the 1960-80s. Borg never pretended the relationship to be linear. On the opposite, he already demonstrated that the relationship was non-linear with ratings of perceived exertion (RPE) intensity varying as a function of (F-b)^n^ where F is the objective force exerted, b a constant and n a power parameter above 1 (see e.g. Borg 1961; Stevens & Cain 1970) suggesting that the relationship is instead exponential, as suggested by the results of the authors of the current manuscript. This result was also confirmed across a wide range of tasks involving to produce physical forces, including handgrip squeezing, but also with bicycle running or walking (see Borg, 1978; Marks & Cain, 1972). More recently, a vast number of studies has also confirmed that the subjective perception of effort is an exponential function of force intensity with paradigms using handgrip forces (see e.g. Hartmann et al, 2013; Klein-Flügge et al, 2015; Le Bouc et al, 2016; Chong et al, 2017; Arulpragasam et al, 2018). Not citing this work leaves the reader with the wrong impression that this result is something new discovered by the authors when it should be really clear that this is widely known for decades now.
  - Related to my previous comment: I found the figure 2 and the associated analysis not very easy to understand. I would suggest to add a Figure 2A before, or to replace it completely, by a figure showing the produced force in % of MVC in the y-axis in function of the instructed/targeted force in % of MVC for each single level. That would greatly facilitate to convey the message that there is not a linear mapping between objective force and RPE and would make the parallel with other studies easier.
  - Also related to that previous comment: it would be nice to 1) fit a linear model for force produced = f(RPE) and 2) to compare it with two non-linear models involving a parabolic or a hyperbolic fit to see which model explains the data better. This can be done in Matlab or in R. I believe that this would be a much stronger point than the 2-by-2 comparison of deviate MVC performed by the authors.
- Concerning claim 2:
  - Of course, the fact that physical fatigue will have an impact on the mismatch between RPE and objective force is widely known and expected and the authors replicated it. When they present this in the discussion “we found that producing force at constant RPE-levels led to a decline in objective force production over time.”, it should be again clear that this is not something new but a replication of a widely expected result.
  - However, interestingly, the authors show that this happens only for the low level of effort (20 and 40%) but not for the high levels of effort (60 and 80%), a phenomenon that the authors attribute to boredom for the low effort levels. However, I suspect that this may also be very sensitive to the way the task was instructed. Was there some instruction that could have biased them to maintain performance for high more than low levels of effort? Was it clear for the participants that they would be paid a fixed 20€ amount at the end, independent of their performance? If not, they could have believed that final performance was relying on their performance and then they could have decided to maintain performance in the high levels more than in the low levels out of strategic thinking. A clarification for the instructions given by the experimenters would be helpful to know if some hypotheses explaining this result can be ruled out or not (boredom vs strategic maintenance for high efforts). Alternatively, it would be interesting to see if this result can be replicated across other datasets exploring physical fatigue in the future but this is not necessary for the current study.
- Claim 3: Based on the EVC model of cognitive control allocation and on the idea that effort allocation is based on a limited and quickly depletable resource, the authors were expecting that the combination of physical and mental effort expectation would induce an increased perception of effort, and therefore a lower force produced because cognitive control, as a limited resource, would have to be allocated to both the physical and the mental effort task. They expected that this effect would even be more pronounced for higher mental effort (2-back) in comparison with the low mental effort (1-back). However, they found the opposite result: participants actually have an increased physical force production when there is both physical and mental effort anticipation, at least at the beginning of the experiment, and this tends to normalize over time. I have several comments on that regard:
  - The authors made a lot of premises that are not consensual for everyone to build up this claim. It should therefore be stated in the introduction clearly that these are choices made by the authors but that those topics are still under much debate in the field. More specifically, 1) they consider that EVC theory is valid and explains well what the brain is doing although this is still widely debated (see (Vassena, Holroyd, Alexander, 2017) and (Clairis, Lopez-Persem, 2023)), 2) EVC can explain physical investment as well as mental investment (this has been claimed in several theoretical papers but no clear proof has been given until now as far as I am aware) because both would be based on the same cognitive control resource, and 3) cognitive control is based on a limited resource and can be very quickly depleted, ie allocating it to physical effort will impede the subsequent allocation to mental effort and therefore subjects would allocate less physical effort to preserve resources for the subsequent mental effort. The disappointment that the authors seem to feel for the fact that their initial hypothesis for claim 3 was not confirmed may in fact just be a reflection that some of these premises are not valid which should be developed a bit in the discussion. Especially the 3^rd^ premise is very linked to the ego depletion theory which has received a lot of criticisms in the past decade, suggesting that cognitive resources may not deplete at least in such fast scales. (see e.g. (Carter, Kofler, Forster, McCullough, 2015) or (Vadillo, 2019)). Moreover, if one forgets about the 3^rd^ premise, the results are not necessarily incompatible with the EVC view of physical effort that the authors are trying to promote in the current paper. One very easy explanation could be that when anticipating both physical and mental effort exertion, people invest more cognitive resources (cognitive control) and therefore they perform better in the physical task at the beginning of the experiment, an effect which goes away slowly as fatigue or boredom arises with time-on-task, at least for the low effort condition.
  - More generally, it could be that when mental effort has to be exerted, people are more aroused because they expect to face a more difficult challenge than in the “no-mental effort” condition which leads them to also produce more force in the physical effort task. In any case, I found the discussion a bit weak on that aspect, as if the authors were just disappointed that their initial hypothesis was not verified which made the paper sound a bit disappointing, when they could highlight their results as interesting and relevant for the field. One major problem here stems from the fact that, since authors did not actually implement an EVC model to their data, it is hard to validate or invalidate this account based on their data. It would be important that authors rewrite the study with this comment in mind in order to reconsider their initial hypothesis along those lines and highlight their results more positively.
  - Again, having a better view on how the task was instructed would be important here. As the authors pointed, reward may play an important role and it was not manipulated here. If the subjects expected final payoff to be higher when they performed better in the hard conditions (based on a prior belief), they may have put more effort when the task was actually harder because of expecting to be more rewarded for it rather than because of the task in itself. Moreover, the authors explain Page 12 that “After each block, performance feedback was given regarding the accuracy of responses across all mental tasks from the preceding experimental block.”. If that was disclosed to the participants in the instructions, they may have felt a pressure to perform better in the mental effort task because they wanted to have positive feedback at the end of each block. This could have led them to invest more attention and effort in the trials associated to mental+physical vs physical task alone in order to maximize the reward part of the EVC considered as positive feedback at the end of each block.
  - Given the strong claim of the authors regarding the fact that participants may have felt bored for low efforts and the global boost observed when mental efforts were required, I was wondering whether participants could have a strong “need for cognition” which would explain the boost in the mental condition (see for example (Zerna, Scheffel, Kührt, Strobel, 2023) and (Maekelae, et al, 2023)). Was there any measure of the level of education of the participants? Maybe this or any other measure that could serve as a proxy for the need for cognition could help to understand the boost observed in the mental effort condition.
  - Figure 3: highlighting the information that is relevant for the reader would be helpful. Right now, it is hard to know where to look and where is the relevant information. An easy way could be to add dotted lines on the center and right panels to show where the initial force level (for the no-mental effort condition) lies for each RPE level, therefore facilitating the comparison. Currently, it’s almost impossible to compare the curves because of the size of the figure.
- Force integration over 2s:
  - The authors report that the force used for calibration was just based on a peak force as is classically done in these type of experiments, but the force required during the task was based on an average over 2 seconds. What was the reason for this procedure and not using a peak force as well? It should be clearly acknowledged in the manuscript as it seems a bit awkward to me to not use the peak force as well in their analysis, which would also probably strongly reduce the delta between force produced and target force which is relatively gigantic right now (range of produced force is between 0 to 35%, while the required range was between 20 to 80%) because of the way produced force is computed.
  - Was there any calibration before the task to ensure that the participants understand what each level meant in terms of force production or are the authors relying on the fact that this should completely rely on their subjective feeling? Still, it seems strange that 80% of perceived exertion does not map above 35% of actual produced force. That would mean that any force produced above 35% of MVC would already feel extremely effortful (>80%) for the participants. It is possible, but it would be nice to 1) control that this is not due to the way force was extracted (not a peak but 2s average) and 2) that this is not due to the fact that participants are not incentivized to perform the task as final payment is fixed, so they could overrate how some force levels feel because they lack any incentive to produce force.
  - Maybe I missed it, but it wasn’t clear to me if the participants get any feedback at all regarding the “true” force required to reach the corresponding levels during training. It seems to me it’s not the case, but it depends on whether the “practice trial” included feedback or not. If there was feedback, there is a high risk that people are closer to the feedback at the start of the experiment and just deviate from it with time-on-task. If not, then that would explain why the actual force is so far from the required force but it would be important to state it clearly in both cases.
- The mental effort conditions, both low and high, imply that subjects perform task switching regularly, a process known to be related to mental effort in itself, while the no-effort condition is just pure physical effort all along. Did the authors consider that in their analysis? Maybe a first analysis grouping low and high mental effort vs no-mental effort could be worth already to sit if there is a global effect of mental effort (task switching + N-back task) vs no mental effort. This is also important because the jump from no-mental effort to low-mental effort could be higher than from low-mental effort to high-mental effort, because of the task switching addition in the mental-effort condition and the possibility to rest (and therefore recover) in the no-mental effort condition. Plus the mental effort condition receives a feedback at the end of each block and not the other condition.

Minor comments

- The introduction, although very well written, clear and enjoyable to read, seemed a bit too long and off the topic of the article, with a long development on the expected value of control (EVC) which is not really implemented (at the computational level). I would center it a bit more on the main questions addressed in the manuscript with a clearer explanation of the premises of the authors (see previous comments).
- The authors use the word “effort” to designate both the level of force produced (ie physical work or force produced) and the subjective feeling that accompanies it (effort). A clear distinction and definition of these two terms could be helpful to improve the clarity of the manuscript (see (Wolpe, Holton, Fletcher, 2024) and (Bermudez, Massin, 2022)).
- Replacing the RPE levels 2/4/6/8 by the % of force (20/40/60/80%) in the manuscript and especially in the figures could help to improve the readability of the manuscript, especially since based on the authors the instructions were “how hard am I squeezing this handgrip dynamometer, relative to my maximum strength?” (Page 18) and that “To facilitate understanding, participants were presented with the modified CR-10 scale, which displayed the respective percentages (20%, 40%, 60%, 80%) next to the target levels (2, 4, 6, 8).” (Page 11).
- The authors decided to use “occurrence” to model the effect of time on the produced force. Did they also tried to use trial number instead? Since trials are interleaved, trial number might be more relevant as it should reflect cumulated fatigue. A nicer way to deal with fatigue though of course would have been to use a more complex model like in (Matthews et al, 2023) implementing recoverable (in the no-mental effort condition and between blocks) vs unrecoverable fatigue (accumulating over all trials) but a first test with trial number would already be interesting.
- Page 4: “During task performance, the EVC is constantly monitored, updated, and adjusted if needed.” Based on what are the authors making that statement? As far as I know, there is not much evidence proving that for now so I would not make strong statements about the EVC computation being constantly monitored, especially since this does not appear to add anything relevant for the current study.
- Page 8: Inclusion/exclusion criteria are missing. The authors just state that participants had to speak German and to not have any arm injuries. What was the age range for the study? Were there any other criteria (related to pathologies for example)?
- Page 8: The data was apparently acquired between 11/20 and 07/22, was there any covid-related information that would be important to reveal? Like were the participants wearing a mask during the experiment (possibly impacting respiration and effort production) or not? And if so, was it the case for all of them or only a subpart? This is not essential but it would be nice to acknowledge.
- Page 9: when the measure of MVC is introduced, this sentence “whereas the measured force for the submaximal contractions was averaged over two seconds” should make it clear that it’s talking about the measured force “in the task”.
- Page 12: “After data acquisition, handgrip measurements and experimental instructions were merged and down-sampled to ten rows per second.” This sentence is unclear. It should probably state “handgrip measurements and experimental instructions periods” but it would probably be easier to explain if the authors give a name to each timeperiod in Figure 1 and then refer to it here.
- Page 12: “The experiment began after completing a practice trial, which could be repeated as often as necessary.” What was this practice trial consisting on exactly? Just squeezing the handgrip? Did they get any feedback or not? Was there any reason for the experimenter to ask the subjects to repeat this practice trial or is solely based on the participants’ willingness to explore how the grip works?
- Page 13: how was mental effort encoded? Was it as 0/1/2 levels corresponding to no/low/high mental effort? The description is clear for the physical but not for the mental.
- Page 14: “Interestingly, no significant differences were found between mental (M = 4.10, SD = 1.98) and physical (M = 4.47, SD = 2.08) exhaustion ratings, indicating that the procedure caused a moderate overall fatigue level.” I understood that fatigue ratings were asked regularly before and after each of the six blocks so it would be nice to clarify what this measure is referring to. Is it just the final fatigue rating? What would be more meaningful would be the delta between the final and the initial fatigue rating for each dimension (ie compare [final fatigue rating-initial fatigue rating] for mental vs physical) which is not the final state but how much the task increased the fatigue ratings.
- Page 22: “Current research argues that force accuracy between intended and actual force can be increased by visual feedback (Kim et al., 2021).” The authors could also expand a bit on the fact that individuals vary widely in their metacognitive and interoceptive capabilities. Therefore, strong interindividual differences could also be present across the participants studied based on this.
- It is known that males generally have a higher strength and MVC than females. It would be interesting to verify whether the main results reported in the study are also valid when one splits the data between male and female just to be sure that differences in capacity and/or in sex do not influence the results presented, but this could go in the supplementary material.
- Was there any measure to prevent participants from doing a force below their actual MVC during calibration or, at least, to debrief at the end of the experiment if they understood that this procedure was to calibrate their MVC on which the rest of the task would be based? In these tasks, sometimes participants understand that this is a calibration and they perform below their actual MVC to make the task easier for them. The answer to my previous comment regarding the instructions for the final reward would also help to answer here as, if they know that the final payment is fixed, they also have no reason to cheat their MVC calibration, but this is an important detail.
