# Supplementary material for: Examining the alignment between subjective effort and objective force production
Source: PLoS One. 2024 Aug 9;19(8):e0307994. doi: 10.1371/journal.pone.0307994 (PMC11315346; doi:10.1371/journal.pone.0307994)
Supplement: Attachment — Submitted filename: Response to Reviewers_ExaminingAlignment.docx [file pone.0307994.s002.docx]

Dear Dr. Akaishi,

#### we would like to thank you and the two reviewers for your time and the very helpful and constructive evaluation of our manuscript. Enclosed you will find the carefully revised second version of the manuscript "Examining the Alignment between Subjective Effort and Objective Force Production”and a point-by-point response to the concerns raised and suggestions made. Responses are in bold blue font, and major insertions to the manuscript are highlighted in italics in the response letter (only visible in the attached letter, not in the online form). Additionally, insertions to the manuscript are highlighted by track changes. We also added the requested clean manuscript version.

We are very much looking forward to your feedback.

**Sincerely**,

**Katja Rewitz;** Dynamics of Human Performance Regulation Laboratory, Insitute of Movement Science, University of Hamburg, Germany

[katja.rewitz](mailto:katja.rewitz)@uni-hamburg.de

**Wanja Wolff**; Dynamics of Human Performance Regulation Laboratory, Insitute of Movement Science, University of Hamburg, Germany

[wanja.wolff@uni-hamburg.de](mailto:wanja.wolff@uni-hamburg.de)

**Sebastian Schindler;** Institute of Medical Psychology and Systems Neuroscience, Münster, Germany, [Sebastian.Schindler@ukmuenster.de](https://sogo.uni-konstanz.de/SOGo/so/pop520330/Mail/view)

**Review Comments to the Author**

**Reviewer #1: The study is interesting and well-conducted, and certainly worthy of publication. Yet, I have identified several areas that require further consideration and clarification:**

**Thank you for the positive overall evaluation!**

**1. In the abstract, while well-written, you present the results as somewhat binary. It would be beneficial to elaborate on the magnitudes of these inconsistencies and their meaningfulness or triviality.**

**We agree that the prior version of the abstract was not comprehensive enough with respect to what we specifically found and who this might be meaningful for. We have now rewritten this according to your comment.**

“[…] We found that the RPE-force alignment was not the same across RPE-levels: *Whereas subjective differences from 20-40% and 40-60% were met by comparable differences in produced force, a substantially larger difference was observed for the 60-80% interval. Interestingly, exploratory post-hoc analyses revealed that this was mirrored by an increase in variance at the higher effort levels*. In addition, at constant RPE-levels, participants produced less force over time, and *this* *effect was more pronounced at lower RPE target levels.* Lastly, anticipating mental effort after the physical effort slightly altered the alignment as a function of the to-be-produced RPE-level and experimental duration. Taken together, our results indicate that the mapping of perceived effort on objective performance is intricate, and several factors affect the degree and shape of how RPE and performance align. *Understanding the dynamic adjustment of RPE-performance alignment across different RPE levels is particularly relevant for contexts that use RPE as a tool for training load prescription.“*

**2. Under the introduction, defining "mental exercise" briefly would enhance clarity, and it's unclear why it's italicized**.

**Thank you for pointing out that “mental exercise” as a term may not be clear in this context. Here, we use “mental exercise” as a counterpart to physical exercise. The term is more commonly found in lay than scientific literature, so we initially decided to italicize it. By “mental exercise”, we refer to any engagement in cognitively stimulating activities. Because we do not want to imply that mental exercise is a scientific term, we now clarify this by explicitly stating that mental exercise is a “lay term” and explaining what we are referring to in the text.**

“Physical exercise is associated with a reduction of health-related risk factors, higher quality of life, and reduced mortality (Chastin et al., 2019; Hummel et al., 2022; Hupin et al., 2015). *Mental exercise is a frequently used lay term describing the engagement in mentally stimulating activities and* is suggested to contribute to developing a "cognitive reserve"*. This reserve is* posited to be a protective factor against cognitive decline and clinical symptoms in the early onset of neurological diseases such as Alzheimer's (Pettigrew & Soldan, 2019; Stern, 2009).”

**3. The extensive review of cognitive control theories might not align with the study's focus, as it revolves around perceived effort and behavioral output alignment.**

**Thank you for highlighting that an extensive review of cognitive control theories might not be necessary for our study’s focus. We agree and have, therefore, shortened the review. To facilitate understanding of our assumptions and hypotheses, we stick to the EVC-theory as an example of effort allocation as a value-based decision and potential changes in effort investment associated with different effort demands or changes in perceived costs over the course of the study. However, we now clarified that most reward-based choice theories in motor and cognitive control research make predictions that are similar with respect to their key premises (that are relevant for us). Namely: Effort carries a cost that people try to minimize and only exert to the degree that it is justified by value/reward. The substantially shortened section now reads:**

“Cognitive control, also referred to as self-control, is defined as "the set of mechanisms required to pursue a goal, especially when distraction and/or strong competing responses must be overcome" (Shenhav et al., 2013, p. 217). Applying control is perceived as effortful (Cohen et al., 1990), and perceived effort is understood to index the costs of applying control (Shenhav et al., 2017). *In the present study, we adapt the definition of perceived effort as “the instantaneous experience of investing resources to perform an action” (Halperin & Vigotsky, 2024, p. 8). In line with this definition we understand the perception of effort to be based on the integration of various contributing factors rather than being solely contingent on objective behavioral output (e.g., force production) (Halperin & Vigotsky, 2024).*

*Motor control and cognitive control theories suggest that the execution of a control action reflects a reward-based choice that is geared towards minimizing control costs (Wolpert, 1997; Shenhav et al., 2013). To illustrate, according to one influential framework—the Expected Value of Control Theory (EVC theory)—the expected value of control is calculated for all available behavioral options by discounting the expected positive and negative payoffs that are associated with task engagement and the intrinsic cost of control. These intrinsic costs scale with task demands and task duration (Shenhav et al., 2013). Thus, harder and longer tasks are expected to be more costly than short and easy ones (Wolff et al., 2021). During task performance, environmental information (e.g., feedback, opportunity for rewards) and internal sensations (e.g., pain, motivational loss, boredom) are assumed to be monitored, updated, and integrated to initiate adjustments in effort allocation (Geana et al., 2016; Shenhav et al., 2013; Shenhav et al, 2016). Consequently, if changes in perceived control costs alter this cost-benefit analysis, performance decrements or even task termination are expected to ensue (Shenhav et al., 2013).*

Empirical evidence demonstrates that the regulation of mental and physical effort can be effectively described by theories relying on the concept of a cost-benefit analysis (Manohar et al., 2015; Wolff et al., 2020). However, recent theoretical and empirical advancements notwithstanding, the relationship between perceived effort as an index of control costs and the translation of this perception into objective behavioral output is not fully understood. Simply put, we do not know how perceived effort levels and objective output align and what factors affect this alignment. In this study, we will investigate these questions through the lens *of reward-based choice theories of control.”*

**4. The rationale for providing feedback on the mental task needs to be clarified**

**Thank you for pointing out this omission. We have now clarified this:**

“After each block, performance feedback was given regarding the accuracy of responses across all mental tasks from the preceding experimental block. *Feedback was provided as a self-monitoring device so participants could make sure that they were correctly performing the different mental tasks throughout the 2-hour experiment.* The feedback provided was not tied to specific trials. Hence, participants were not given any information on their performance regarding different task difficulties.”

**5. The setup of the experiment is a little confusing. Were the X% randomized? Did, for example, all reps with 20% come in one block? Please clarify this point.**

**Thank you for asking. Conditions were randomized. Thus, not all reps with 20% were performed consecutively. We clarified this in the methods section.**

“Participants were asked to produce four force levels in alignment with predetermined target levels of a modified Borg CR-10 scale (Borg, 1982). The Borg CR-10 scale is a 10-point category-ratio-scale used to measure exertion and pain (Capodaglio, 2001). The upper anchor of 10 represents the maximal possible exertion. The target levels 2, 4, 6, and 8 were selected to cover nearly the whole scale range. Participants were instructed that these levels should represent 20%, 40%, 60%, and 80% of their maximum. *The target levels were presented in a random order throughout the experiment.* During the whole experiment, participants performed 240 submaximal contractions.”

**6. In the statistical analysis section, it's unclear what the outcome was for the one-way ANOVA. Clarifying whether it was the differences in forces between those produced and calculated based on the MVC is necessary**.

**We fully agree that this was not worded clearly enough in the prior submission. We have now provided a more precise description of what was tested in the ANOVA. This has clarified our reasoning and intended outcome.**

“To investigate whether the subjective *differences of the RPE scale intervals transform into the same intervals in objective force*, a one-way repeated measures ANOVA was performed with the *averaged differences in produced force (expressed in %MVC) between target RPE levels as the dependent variable.* Thus, *we compared whether* the relative difference in produced force for the three equal-sized subjective intervals of effort (i.e., 20-40%, 40-60%, and 60-80%), each reflecting a 20% increase in subjective effort, *translate into equivalently sized intervals in produced force.”*

**7. In the results section, you should acknowledge the impact of the normalization procedure on the low values (i.e., mean normalized to peaks).**

**Good point! We have updated the manuscript accordingly:**

“In physical effort level 2, force production differed *M* = 11.19% (*SD* = 6.66%), in level 4 *M* = 24.93% (*SD* = 8.59%), in level 6 *M* = 39.31% (*SD* = 10.53%) and in level 8 *M* = 46.86% (*SD* = 14.18%) from target level MVC. *This underproduction relative to peak MVC is likely due to the fact that target level MVC was calculated as a proportion of the achieved peak force, whereas force production was averaged across 2 seconds.”*

**8. Under “Alignment of subjective effort level and objective force,” Maybe add the actual values in the text rather than just on the graph. It seems as if the error (i.e., RPE-based forces – calculated % based on MVC) is around 5% for the 2-4 and 4-6 conditions and increases to roughly 10% for 6-8. There is little discussion over these values, yet I think they are very important.**

**Thank you. We added the actual values in the text to make the non-equivalent differences in produced force between RPE target intervals easier to understand.**

“[…]A significant difference was found when comparing the averaged deviations between the RPE-level intervals (*F*(1.36, 57.14) = 63.85, *p* < .001, $\eta_{g}^{2}$= 0.34). *The deviations represent the differences in the averaged produced force (in % MVC) between target level 20% compared to 40% (M = 6.26%, SD= 3.18%), 40% compared to 60% (M = 5.62%, SD = 3.74%) and 60% compared to 80% (M = 12.45%, SD = 5.58%).* Bonferroni corrected post-hoc analyses revealed a significant difference in produced force relative to MVC between the target levels 20% and 40% compared to 60% and 80% (*t*(42) = -7.43, *p* < .001) and between the target levels of 40% and 60% in comparison with 60% and 80% (*t*(42) = -9.92, *p* < .001). The deviation between the 20% and 40% target levels did not differ from the deviation between the target levels of 40% and 60% (p = .39).[…]”

**9. The discussion section could benefit from shortening the second paragraph and considering another term for "equidistant." Additionally, discussing the magnitude of the error and its importance would enhance the interpretation of the findings.**

**We agree that expanding on the increased variance at higher effort levels is important here! We have now added an analysis of the error variance to the paper (clearly marked as an unplanned exploratory post-hoc analysis) and we now discuss this in the second paragraph of the discussion. Particularly, we discuss what this might mean for applied settings. Our reading of this finding is that prescribing intensity at low to moderate RPE levels should lead to relatively consistent force production (due to lower variance), whereas an exerciser might produce vastly different forces at high subjective effort levels (due to higher variance).**

**(Regarding the term ‘equidistant’: we have considered another term for this but after careful deliberation and also looking for other terms in the literature, we would prefer to keep using the term. This is because we feel it accurately describes what we refer to and because it is used in other studies too. However, we are clearly not ‘married’ to it, so if you insist on it being not the adequate term here, then we are happy to change it in another round of revisions)**

**Thanks again for pointing us towards the relevance of looking into the errors more:**

“[…]*Error bars in Figure 2 indicate that the variances in produced forces are not the same for each effort level. Consequently, we conducted an unplanned exploratory post-hoc analysis to assess whether variances differed significantly between levels. Consistent with the descriptive trends in Figure 2, a repeated measures ANOVA and post hoc comparisions revealed that variance increased at higher effort levels F(1.32, 55.26) = 67.96, p < .001), with a significantly higher variance at target level 80% compared to the lower effort levels (p < .001).* For the full analysis, please see [*OSF | alignment subjective effort_objective force production*](https://osf.io/9dyxn/?view_only=081a9f5000e7413d81fcbc749e9c4c64)*.”*

**“**First, we found that equidistant RPE-levels do not map directly on equidistant handgrip force. In line with previous research, equidistance was found at lower effort levels (i.e., same size differences in objective force when comparing 20-40% with 40-60%) but not at high effort levels (i.e., a larger difference in objective force in the 60-80% comparison when contrasted with the 20-40% and 40-60% comparison)*(Cochran et al., 2007; Pincivero, 2011). The difference in produced force between a perceived effort of 60% and 80% was twice as much as the difference between the lower RPE intervals (20-40, 40-60), indicating that different factors contribute to the perception of effort at different levels of the RPE scale. This finding is consistent with other research that has found effort perception and produced force to be not aligned in the same way across all effort levels. Interestingly unplanned exploratory post-hoc analyses revealed that at higher effort levels force production was more variable. Descriptively, the pattern mirrored the pattern found with respect to the mean forces, indicating a markedly increased variance at the 80% level. Taken together, these results point towards marked differences in how subjective effort aligns to objective performance at the upper end of the RPE scale: alignment seems to be rather straightforward and consistent at low to moderate subjective effort levels, and this mapping becomes much more variable at high effort levels. From an applied perspective, this implies that prescribing intensity at low to moderate RPE levels should lead to relatively consistent force production, whereas an exerciser might produce vastly different forces at high subjective effort levels.“*[…]

**10. Discussing the (mis)alignment should also consider directionality. If subjects produced more rather than less force, then I fail to see how opportunity costs or pain, for example, are a good explanation.**

**Yes, we agree that opportunity costs are not a very plausible explanation for overproduction. In the prior version of the manuscript, it indeed reads as if we were discussing the relative overproduction at higher effort levels in terms of opportunity costs. In an attempt to remove this source of misunderstanding, we have now restructured this part of the discussion. When discussing the potential reasons for relative overproduction at higher levels, we now primarily discuss differential recruitment of type 1 and type 2 muscle fibers (this might actually also be an interesting explanation for the higher variability at higher levels in our point of view). We then discuss the effects of opportunity costs in general – and boredom in particular – in the next section where we discuss potential reasons for the drop in force at lower levels over time. We think this makes this section much clearer. The respective parts now read as follows:**

**Overproduction (no opportunity cost/boredom interpretation)**

*Reward-based choice theories claim that* physiological and psychological variables *likely* contribute to differential increases in effort costs and the attendant differences in produced force. With respect to physiological variables, research shows, for example, that muscle fiber recruitment differs across effort levels (Gregory & Bickel, 2005; Miyamoto et al., 2020). *One speculative interpretation of the relatively large difference in force production from 60%-80% might reflect the additional recruitment of Type 2 muscles fibers, thereby altering the perceived effort costs at higher levels. Differential muscle fiber recruitment might also affect the precision with which people can produce force according to their subjective perception of effort, as research shows that type 1 fibers allow for more graded and controlled contractions (e.g., Henneman et al., 1965; Moritani, 2003).* Through this lens, it is plausible that the alignment across effort levels might not be equivalent.

**Temporal decline only at lower level (boredom interpretation)**

“This reasoning can be extended to the observation that produced force declined over time and that this decline depended on the required level of subjective effort. Through a *reward-based choice lens*, changes in physiological (e.g., reduced actual capacity as reflected in a lowered MVC) and psychological states (e.g., increased boredom) *should affect perceived momentary control costs in a highly dynamic fashion*. For example, MVC is expected to decline over repeated submaximal contractions over time (e.g., Taylor & Gandevia, 2008). One tentative explanation for the lower forces we observed over time could be that participants dynamically adjusted their efforts according to the gradual decline in MVC. Importantly, force declines were only observed at subjective efforts of 20% and 40% but not at 60% or 80%. This suggests *that a gradual reduction in MVC was not the only reason for lower force production over time* and that additional factors *might also affect this decline.* One speculative interpretation for the force decline at lower intensities might be that easy trials felt more boring than the higher effort trials. *Consistent with this research has frequently linked* the experience of boredom to underchallenge in monotonous tasks (Eastwood et al., 2012; Markey et al., 2014). *In addition, recent work on physical effort showed that low effort tasks can feel more boring than high effort tasks (Rewitz et al., 2024).* *In addition, unpublished work from out lab indicates that people feel that they have to exert additional effort due to boredom. Unfortunately, this interpretation requires future research because we did not track boredom throughout this experiment.*

**11. A limitation that should be mentioned is the lack of post-protocol MVC, or better yet, MVCs performed throughout the protocol to better assess the error rate, given that MVCs were expected to change. Based on your results, it may be that subjects are able to make more subtle adjustments with lower RPEs than higher RPEs, since they produced lower forces over time, which aligns with the sensible notion that fatigue played a role and that their MVCs were reduced.**

**We added this to our limitation section! Indeed, this would be a better marker of the relative deviation of the subjective MVC at that specific moment in time. However, due to the randomization procedure, we still see a relative difference in behavior across the four effort levels, meaning that irrespective of temporal changes of the MVC, the relative change cannot be explained by a temporal decrease of subjective MVC (see also above concerning mini-block randomization).**

*“Finally, we did not measure MVC levels throughout the experiment to assess the error rate better, given that momentary MVC may change over time. It may be that subjects can make more subtle adjustments with lower RPEs than higher RPEs since they produced lower forces over time, which aligns with the notion that fatigue played a role and that their MVCs were reduced. Importantly, in this study, we were interested in how subjective effort differences robustly translate into objective differences in produced force. As the to-be-produced forces were prompted in randomized order, the relative differences between the four levels are unlikely to be driven by the temporal changes in MVC.”*

**Thank you for your feedback and the opportunity to revise our paper. We appreciate your comments and suggestions and have implemented them to enhance the quality of our manuscript.**

**Reviewer #2: The review has been added as an attachment because it exceeds 20,000 characters. Main message is copied below but please refer to the attached document for more details.**

**The manuscript has two main focuses. One is to study the mismatch between objective and subjective force exertion and how this mismatch can be altered by fatigue/time-on-task. The second focus is to build upon the idea that physical effort depends on the expected value of control (EVC) model developed by Shenhav, Botvinick and Cohen in 2013, as has already been suggested in previous publications, and to test whether effort is allocated differently when one anticipates to exert only physical effort versus when one anticipates to exert both physical and mental effort, with the idea that both could share a limited and quickly depletable resource. The three specific claims are that 1) subjective ratings of perceived exertion (RPE) are not matching objective force intensity linearly in a handgrip squeezing task, especially for high (60-80%) force levels, 2) time induced a reduction in the objective force exerted over time for constant RPE levels, and 3) that there is an interaction between time-on-task, and the anticipation of physical effort alone vs the anticipation of physical and mental effort.**

**Overall, I found the article well-written and easy to follow and read. I think that some of the results are quite interesting, either because they replicate some results (claims 1 and 2) with some new results specific to their study (claim 2) or because they discovered something new that could reflect interesting processes about how the brain proceeds with effort investment (claim 3). However, I think that the current version has some major flows, especially regarding the methodological description and the way the results are presented, and I believe that a major revision could help to significantly improve the quality of the paper.**

**Thank you for your thoughtful review and feedback on our study. We appreciate the opportunity to address the points of criticism.**

**Major comments**

**1. The authors seem to put the three claims on the same scale, but a vast literature already exists for claim 1 that the authors seem to ignore or to set aside. I think that the manuscript should not put the three claims on the same level to highlight what is in the order of replication and what is really new in their study.**

**Concerning claim 1:**

**the authors present the fact that there is a nonlinear relationship between objective and subjective force exertion as something new, or at least they omit to mention that this is widely known for decades. However, this result was already presented in the initial work from Gunnar Borg, that the authors cite already in the manuscript, in the 1960-80s. Borg never pretended the relationship to be linear. On the opposite, he already demonstrated that the relationship was nonlinear with ratings of perceived exertion (RPE) intensity varying as a function of (F-b)^n^ where F is the objective force exerted, b a constant and n a power parameter above 1 (see e.g. Borg 1961; Stevens & Cain 1970) suggesting that the relationship is instead exponential, as suggested by the results of the authors of the current manuscript. This result was also confirmed across a wide range of tasks involving to produce physical forces, including handgrip squeezing, but also with bicycle running or walking (see Borg, 1978; Marks & Cain, 1972). More recently, a vast number of studies has also confirmed that the subjective perception of effort is an exponential function of force intensity with paradigms using handgrip forces (see e.g. Hartmann et al, 2013; Klein-Flügge et al, 2015; Le Bouc et al, 2016; Chong et al, 2017; Arulpragasam et al, 2018). Not citing this work leaves the reader with the wrong impression that this result is something new discovered by the authors when it should be really clear that this is widely known for decades now.**

**Thank you for your careful review of the literature. We agree that our introduction was not written clearly enough. Especially with respect to the novelty of the alignment question and the claims made by different researchers about the function of alignment. We did not intend to suggest that Borg claimed that perceived exertion would map in a linear fashion on objective output measures. As you mentioned, Borg’s research consistently points out that while the Borg scales are category-ratio scales, structured in a linear manner, various perceptions are integrated into the perceived exertion. In turn, RPE represents a fascinating compound measure that should probably not be expected to linearly map onto one singular marker of performance (importantly, in our reading of the literature this linearity is often assumed, for example when the “validity” of the scale to capture certain physiological dynamics is discussed in estimation settings (e.g., Chen et al., 2002, Lea et al., 2022). We fully agree with your claim that this is not actually implied by the scale itself and this is also what has driven the present research: to study how RPE production translates to one measure of performance. We have now clarified this on page 5.**

**Most studies that suggest that the the alignment follows an exponential and not a linear function used the magnitude estimation method. Some studies use a production method but rarely are solely instructed with an RPE scale. Often, participants first rate executed forces and then produce forces corresponding to their rated RPE. However, we are interested in what happens when participants produce forces instructed by percentages relative to their maximal capacity, a commonly used practice in sports and rehabilitation settings. Using a category-ratio scale and instructing the MVC as 100%, we would assume linearity in instructed subjective effort levels, such that the perceived effort difference between 20%-40% is equivalent to the difference between 40%-60% and the difference between 60-80%. (Importantly, consistent with your point, we are not stating that the validity of an RPE measure hinges on an equivalent translation of these subjective differences into equidistant distances in an objective performance measure.) Most studies have investigated this question in estimation settings and studies that have used a production method are fewer. In the revised version of the manuscript, we are now making it clearer that we are not the first to study this, but that we are adding to a (still relatively small) body of literature that has investigated the alignment between RPE and objective force in a production setting:**

“Does an increase in perceived effort during a handgrip task lead to an equivalent increase in produced force? Some research *shows that equivalent differences in perception of effort translate to equivalent differences in heart rate (Borg, 1982; Borg & Dahlström, 1959). However, from a conceptual point of view, it is important to note that equivalent differences in subjective exertion do not necessarily need to translate into equivalent differences in objective performance outcomes or physiological indicators of physical strain: Psychometrically, RPE scales are designed as category-ratio scales that measure differences in the perception of effort on an interval scale. However, this perception likely integrates various physical and psychological perceptual cues, and is not solely dictated by a single physiological parameter (Borg, 1962, Borg, 1982). In turn, the alignment between RPE and a singular objective measure of performance might not be linear (e.g., because different additional RPE-determining cues might be integrated at different levels of perceived effort). Indeed, studies that have used performance measures like handgrip strength indicate that the alignment follows an exponential function (e.g., Borg, 1961, Stevens & Cain, 1970, Marcs & Cain, 1972).* *The bulk of this research uses a combination of estimation (i.e., participants are asked to rate their RPE regarding their physical performance) and production methods. Only a few studies exclusively focus on the production method (Chen et al., 2002).* However, RPEs are not only used to ask participants for their perceived effort after stimulus presentation but are also critical for prescribing and monitoring exercise intensity (Hartshorn & Lamb, 2004; Williams, 2017) for example in clinical rehabilitation and sports contexts (e.g., cycling, running and resistance training) (Christian et al, 2014; Helms et al., 2016; Hobbins et al., 2019). This involves a production method where people are instructed to generate force at different subjective effort intensities, with the produced force being the dependent variable of interest. *Instructions for intensity prescriptions are typically relative to a previously measured individual’s maximal capacity, such as “Press 20% of your maximal possible force” (Cochran et al., 2007; Li & Yu, 2011; Morrin et al., 2018; Pincivero, 2011). This practice implies an assumption of a linear alignment between perceived effort and to-be-produced force, with participants using their perceived maximal force as a benchmark and scaling other perceived intensities relative to that (in turn, coaches would likely expect that their athletes train at the specified levels of physical load).* Studies that used such production tasks reported heterogeneous results with respect to the translation of subjective effort into objective performance (Cochran et al., 2007; Li & Yu, 2011; Pincivero, 2011). To illustrate, one study observed that up to 80% of participants' maximum voluntary contraction (MVC), subjective effort, and objective performance were aligned in a linear fashion, indicating equivalence of differences. However, equivalence was not found at higher intensities (Cochran et al., 2007). Similarly, another study found differences in the alignment across effort levels as a function of the study population (Pincivero, 2011). Because of this mixed evidence and relatively few studies using a production method, a deeper understanding of how increases in perceived effort map onto changes in produced force is crucial.”

“[…] We, therefore, *expected to replicate* the non-equivalence in the alignment between subjective effort and *produced* force.[…]”

**2. Related to my previous comment: I found the figure 2 and the associated analysis not very easy to understand. I would suggest to add a Figure 2A before, or to replace it completely, by a figure showing the produced force in % of MVC in the y-axis in function of the instructed/targeted force in % of MVC for each single level. That would greatly facilitate to convey the message that there is not a linear mapping between objective force and RPE and would make the parallel with other studies easier.**

**Thank you for your suggestion to change or replace the current figure. We agree that the figure depicting the deviations and the respective analyses are complicated to understand. Therefore, we have adopted your idea to replace it with a clearer figure displaying the target RPE level and the associated produced force in %MVC.**

**Fig. 2** *Produced force relative to maximum voluntary contraction in each RPE target level*. The difference between each RPE-level represents a 20% increase in subjective effort. As the differences between the compared RPE-levels are equivalent, equivalence in translating subjective effort to objective performance would require a linear increase of produced force across RPE target-levels. *However, the deviation between target levels 60% to 80% significantly differs from the other two deviates (20-40, 40-60), resulting in a nonlinear pattern.* This indicates that equivalent differences in subjective efforts do not translate to equivalent differences in objective force across all effort levels. *N =* 43. Error bars represent the standard deviation

**A) Also related to that previous comment: it would be nice to 1) fit a linear model for force produced = f(RPE) and 2) to compare it with two nonlinear models involving a parabolic or a hyperbolic fit to see which model explains the data better. This can be done in Matlab or in R. I believe that this would be a much stronger point than the 2-by-2 comparison of deviate MVC performed by the authors**

**Thank you for your suggestion to change these analyses. Given our interest in the differences, i.e., the relative changes between the levels, we could only compare linear vs. quadratic contrasts as we only have three equidistant levels (differences levels 20-40%, 40-60%, and 60-80%). While this would certainly be an interesting additional insight for a design that has used more RPE levels, we found this analysis does not substantially add to the questions we are trying to answer with our paper (given our design). However, we agree that investigating different model fits across a higher-resolution design would be a very interesting question indeed. We have added this as an idea for future research to the discussion:**

“[…] *Nonetheless, future research should examine the alignment using all 10 levels to determine whether this entails any changes in the* *shape of alignment*. *For example, it is conceivable that parabolic or hyperbolic functions would better describe the alignment between RPE and objective force, or that the shape of alignment shifts as a function of time (e..g., a linear alignment in the beginning an a hyperbolic fit towards the end).*[…]”

**3. Concerning claim 2:**

**A) Of course, the fact that physical fatigue will have an impact on the mismatch between RPE and objective force is widely known and expected and the authors replicated it. When they present this in the discussion “we found that producing force at constant RPE-levels led to a decline in objective force production over time.”, it should be again clear that this is not something new but a replication of a widely expected result.**

**We fully agree that the observation of a decline in force over time at a given RPE level is not a new finding. We have now revised this section to make clearer where our findings replicate prior research and where they add new insights. Specifically, while it is well known that people report higher RPE levels as fatigue accumulates in estimation studies, it is less well studied how people adjust the alignment between RPE and force in production scenarios over time. More importantly, while adjusting one’s force to a temporal decline in MVC might explain an overall decline in the force people produce across levels, this is not what we found. Since we did not observe a decrease in force at RPE levels 60% and 80%. This suggests that factors other than fatigue accumulated and might influence the decision on how much effort should be exerted at a given time at a given level, leading to a decline in force. We agree that our wording of this finding was not precise enough in the prior version of the manuscript and we have now taken great care to clarify what is a replication and what is a new finding. Thanks you very much for pointing this out!**

"[…] Second, *consistent with other research, we found that producing force at constant RPE-levels led to a decline in objective force production over time (Forman et al., 2022).* Interestingly, this effect depended on the effort participants were asked to produce. When asked to produce a force that reflected subjective efforts of 20% or 40%, the produced force declined over time, *while no such decline was noted for subjective efforts of 60% or 80%.* This indicates that the degree of subjective and objective alignment is sensitive to effort duration and intensity […]"

**And further down we now write:**

"This reasoning can be extended to the observation that produced force declined over time and that this decline depended on the required level of subjective effort. Through *a reward-based choice lens*, changes in physiological (e.g., reduced actual capacity as reflected in a lowered MVC) and psychological states (e.g., increased boredom) *should affect perceived momentary control costs in a highly dynamic fashion.* For example, MVC is expected to decline over repeated submaximal contractions over time (e.g., Taylor & Gandevia, 2008). One tentative explanation for the lower forces we observed over time could be that participants dynamically adjusted their efforts according to the gradual decline in MVC. Importantly, force declines were only observed at subjective efforts of 20% and 40% but not at 60% or 80%. This *suggests that a gradual reduction in MVC was not the only reason for lower force production over time* and that additional factors *might also affect this decline*. One speculative interpretation for the force decline at lower intensities might be that easy trials felt more boring than the higher effort trials. *Consistent with this, research has frequently linked* the experience of boredom to underchallenge in monotonous tasks (Eastwood et al., 2012; Markey et al., 2014). *In addition, recent work on physical effort showed that low effort tasks can feel more boring than high effort tasks (Rewitz et al., 2024). In addition, unpublished work from out lab indicates that people feel that they have to exert additional effort due to boredom. Unfortunately, this interpretation requires future research because we did not track boredom throughout this experiment.*

**4. However, interestingly, the authors show that this happens only for the low level of effort (20 and 40%) but not for the high levels of effort (60 and 80%), a phenomenon that the authors attribute to boredom for the low effort levels. However, I suspect that this may also be very sensitive to the way the task was instructed. Was there some instruction that could have biased them to maintain performance for high more than low levels of effort? Was it clear for the participants that they would be paid a fixed 20€ amount at the end, independent of their performance? If not, they could have believed that final performance was relying on their performance and then they could have decided to maintain performance in the high levels more than in the low levels out of strategic thinking. A clarification for the instructions given by the experimenters would be helpful to know if some hypotheses explaining this result can be ruled out or not (boredom vs strategic maintenance for high efforts). Alternatively, it would be interesting to see if this result can be replicated across other datasets exploring physical fatigue in the future but this is not necessary for the current study.**

**Thank you for your questions. We agree that the absence of force decrements at higher physical effort levels can often be explained by participants belief that higher performances, i.e., higher force productions, are associated with higher rewards. Indeed, people seem to equate “better performance” often with “more effort” (Xu et al., 2023). To address this, we explicitly instructed participants before the study that they would be paid a fixed reward of 20€ and that they would not receive any feedback about their physical performance.**

“A total of *N* = 44 *adult university students* (*f* =18, *m* = 26) took part in the study (*M* = 23.96 ± 2.66 years old, *Min = 18, Max = 32).* *Participants were recruited to participate in a study, where they receive a flat payment of 20€ for their participation.* […]”

“[…] *For force productions participants should solely rely on their subjective feelings. No feedback was provided on either the MVC measures or the submaximal measurements[…]”*

“[…] Importantly, in order to make participants truly rely on their internal sensations of effort, no feedback or monitoring of their physical performance was provided[…]”

*“*[…]*However, the mobilization of effort should depend on how much effort a person feels is justified by the reward value of the task. To make sure that the participants' level of effort was not affected by rewards for good performance, they received a flat payment that was independent of their performance.* *Additionally, we provided no feedback on the quality of their performance in the physical task* […]”

**5. Claim 3: Based on the EVC model of cognitive control allocation and on the idea that effort allocation is based on a limited and quickly depletable resource, the authors were expecting that the combination of physical and mental effort expectation would induce an increased perception of effort, and therefore a lower force produced because cognitive control, as a limited resource, would have to be allocated to both the physical and the mental effort task. They expected that this effect would even be more pronounced for higher mental effort (2-back) in comparison with the low mental effort (1-back). However, they found the opposite result: participants actually have an increased physical force production when there is both physical and mental effort anticipation, at least at the beginning of the experiment, and this tends to normalize over time. I have several comments on that regard:**

**A) The authors made a lot of premises that are not consensual for everyone to build up this claim. It should therefore be stated in the introduction clearly that these are choices made by the authors but that those topics are still under much debate in the field. More specifically, 1) they consider that EVC theory is valid and explains well what the brain is doing although this is still widely debated (see (Vassena, Holroyd, Alexander, 2017) and (Clairis, Lopez-Persem, 2023)), 2) EVC can explain physical investment as well as mental investment (this has been claimed in several theoretical papers but no clear proof has been given until now as far as I am aware) because both would be based on the same cognitive control resource, and 3) cognitive control is based on a limited resource and can be very quickly depleted, ie allocating it to physical effort will impede the subsequent allocation to mental effort and therefore subjects would allocate less physical effort to preserve resources for the subsequent mental effort. The disappointment that the authors seem to feel for the fact that their initial hypothesis for claim 3 was not confirmed may in fact just be a reflection that some of these premises are not valid which should be developed a bit in the discussion. Especially the 3^rd^ premise is very linked to the ego depletion theory which has received a lot of criticisms in the past decade, suggesting that cognitive resources may not deplete at least in such fast scales. (see e.g. (Carter, Kofler, Forster, McCullough, 2015) or (Vadillo, 2019)). Moreover, if one forgets about the 3^rd^ premise, the results are not necessarily incompatible with the EVC view of physical effort that the authors are trying to promote in the current paper. One very easy explanation could be that when anticipating both physical and mental effort exertion, people invest more cognitive resources (cognitive control) and therefore they perform better in the physical task at the beginning of the experiment, an effect which goes away slowly as fatigue or boredom arises with time-on-task, at least for the low effort condition.**

**Thank you for this very thorough and thoughtful comment! We agree, EVC theory is not the only theory to explain control allocation and is not without critique. Importantly, the present paper indeed does not represent a specific test of EVC theory but is rather informed by the general agreement between various reward-based choice theories of control decision: (e.g., Brehm & Self, 1989; Wolpert, 1997); and we use EVC as our exemplary framework) that various control costs exist, that they are integrated over time and that people try to optimize how they spend their resources. We agree that this was not clearly enough expressed in the prior version of the manuscript and we we have rewritten the introduction now in parts to make this more clear.**

**It is important to point out that our third premise regarding the conservation of physical resources when mental demands are anticipated is not based on the concept of the ego-depletion theory. We apologize if this was wrongly implied in the prior version of the paper. Our experimental paradigm is not aimed to reflect a sequential two-task paradigm where a depleting task has a detrimental effect on a subsequent effortful task due to limited metabolic capacities, and with that anticipation, resources are withheld / conserved.**

**Consistent with other theories like, for instance, the Motivational Intensity Theory and the EVC theory, we follow the reasoning that the exertion of effort feels aversive, and effort indexes the costs of control allocation across both the physical and the mental domain. We assumed that in anticipation of a mandatory mental effort task, physical resources are conserved with the aim of still maximizing the expected value of effort allocation. We clarified this in more detail on page 7 to allow the understanding of our mental effort manipulation introduced on page 8.**

“A large body of research has investigated the interplay between physical and mental effort regulation (e.g., Giboin & Wolff, 2019; Holgado et al., 2020; Stets et al., 2020; Wolff et al., 2021). On a conceptual level, this research points towards similarities in the underlying cost-benefit analyses (Manohar et al., 2015; Morel et al., 2017) that govern the decision of whether to employ effort (Marcora & Staiano, 2010; Shenhav et al., 2013) or instead withhold and conserve it (Brinkmann et al., 2021), as well as to similar neurocomputational underpinnings (Ritz et al., 2020; Shenhav et al., 2017). *Simply put, irrespective of the specific effort-domain (physical or mental), people seem to minimize its exertion and make exertion levels contingent on the expected rewards. This begs the question if/how the costs of either effort domain carry-over to the other domain. For example,* researchers have investigated the effects of prior mental effort on subsequent physical performance (e.g., Giboin & Wolff, 2019; Holgado et al., 2020) and the effects of concurrently performing a mental effort task on physical performance (e.g., Stets et al., 2020). *These studies tend to show that prior mental effort can undermine subsequent physical effort and that concucrrent exertion of effort in the mental and physical domain can impair performance.* However, to the best of our knowledge, research has not yet investigated the potential effect of prospective mental effort on the regulation of physical effort. In light of the similarities in how physical and mental effort are governed, it is plausible that prospective efforts in one domain could affect regulation in another domain. For example, a person's effort during a run might be affected by the expectation of having to study for a demanding exam afterwards. As people strive to conserve resources, it is plausible that prospective efforts in one domain affect effort exertion in another domain.”

**However, we agree that the physical and mental tasks could be seen as one connected task. The anticipation of control demands leads participants to invest control in the physical task at the beginning, resulting in higher effort allocations in the force production, particularly at the beginning and mid-experiment, with observed decreases at the end due to other contributing sensations like fatigue or boredom. We discuss that accordingly (see text cite below)**

**6. More generally, it could be that when mental effort has to be exerted, people are more aroused because they expect to face a more difficult challenge than in the “no-mental effort” condition which leads them to also produce more force in the physical effort task. In any case, I found the discussion a bit weak on that aspect, as if the authors were just disappointed that their initial hypothesis was not verified which made the paper sound a bit disappointing, when they could highlight their results as interesting and relevant for the field. One major problem here stems from the fact that, since authors did not actually implement an EVC model to their data, it is hard to validate or invalidate this account based on their data. It would be important that authors rewrite the study with this comment in mind in order to reconsider their initial hypothesis along those lines and highlight their results more positively.**

**Thank you for emphasizing that our results provide promising new insights and should be discussed more clearly. We did not intend to convey disappointment. We acknowledge the significance of our findings, especially considering their potential implications if replicated in future studies. We agree that this was not clearly enough expressed. We have rewritten the section to clarify that. We have also integrated your suggestion regarding potential higher arousal levels into our discussion.**

“Interestingly, we found that the alignment between subjective effort and objective force was only marginally affected by the prospect of having to engage in subsequent mental effort. If participants conceptualize mental and physical effort as one general cost factor per trial, but still see the mental and physical task as two distinct tasks, then we would have expected that subsequent mental effort would prompt participants to withhold physical effort. However, in contrast with our expectations, during the first part of the experiment, participants produced more force when they expected to perform a mental task afterwards. *One potential explanation for this finding might be that the physical and mental task were perceived as one connected task, resulting in a shared effort investment in both tasks.* Consequently, a potential explanation for this finding might be the concept of automatic effort mobilization (Gendolla & Silvestrini, 2010; Silvestrini & Gendolla, 2013). *This concept suggests* that the anticipation of (any) effort and, therefore, an "action prime" leads to increased sympathetic arousal and a higher mobilization of resources compared to when no subsequent effort was required (i.e., an “inaction prime”). *However, the mobilization of effort should depend on how much effort a person feels is justified by the reward value of the task.* *To make sure that the participants' level of effort was not affected by rewards for good performance, they received a flat payment that was independent of their performance.* *Additionally, we provided no feedback on the quality of their performance in the physical task*. *Still, the feedback provided on mental task performance might have served as an incentive, enhancing the perceived rewards in the task configurations where a mental task is anticipated, leading to a higher effort investment.* *One factor that might contribute to higher force production in the trials with an anticipated mental task might be “need for cognition*” (Cacioppo et al., 1996; Mækelæ et al., 2023; Zerna et al., 2023)*. This refers to the tendency to engage in cognitively challenging tasks and university students are assumed to score high on this tendency to value cognitive effort (Cacioppo & Petty, 1982). Thus, one might speculate that the prospect of a mental task trial increased the reward value of the task, thereby licencing a relatively higher investment of effort. However, recent work that has investigated the domain-specificty of peoples’ effort perception showed that high need for cognition is not strongly related to peoples willingness to mobilize physical effort* *(Wolff et al., 2023). Taken together, these results suggest that participants demonstrate a high flexibility in their effort regulation, making fine-graded adjustments based on the integration of various sensations. However, it is important to note that the magnitude of these effects is small, and our results do not indicate that prospective mental effort factors are integrated into the computation of current physical effort costs in a straightforward way.* Further research is needed to assess whether this is because people treat mental and physical effort as separate cost factors or due to methodological constraints in our research design. *In future studies, it will also be be interesting to assess how individual differences in peoples perception physical and mental effort alter the alignment between subjective effort and objective performance across different effort domains.”*

**7. Again, having a better view on how the task was instructed would be important here. As the authors pointed, reward may play an important role and it was not manipulated here. If the subjects expected final payoff to be higher when they performed better in the hard conditions (based on a prior belief), they may have put more effort when the task was actually harder because of expecting to be more rewarded for it rather than because of the task in itself. Moreover, the authors explain Page 12 that “After each block, performance feedback was given regarding the accuracy of responses across all mental tasks from the preceding experimental block.”. If that was disclosed to the participants in the instructions, they may have felt a pressure to perform better in the mental effort task because they wanted to have positive feedback at the end of each block. This could have led them to invest more attention and effort in the trials associated to mental+physical vs physical task alone in order to maximize the reward part of the EVC considered as positive feedback at the end of each block.**

**Thank you for highlighting the potential effects of the provided feedback on mental effort tasks. We fully agree that participants may have directed their attention towards performing well in the mental effort task, especially considering that feedback was provided only for this task. The feedback received could maximize the reward. We discussed that accordingly (see in text cite above).**

**8. Given the strong claim of the authors regarding the fact that participants may have felt bored for low efforts and the global boost observed when mental efforts were required, I was wondering whether participants could have a strong “need for cognition” which would explain the boost in the mental condition (see for example (Zerna, Scheffel, Kührt, Strobel, 2023) and (Maekelae, et al, 2023)). Was there any measure of the level of education of the participants? Maybe this or any other measure that could serve as a proxy for the need for cognition could help to understand the boost observed in the mental effort condition.**

**This a super interesting point – thanks a lot for directing our attention to this. Indeed, all participants were university students. So, while we did not use the need for cognition scale, it is plausible that our participants would be rather high on need for cognition (e.g., Cacioppo & Petty, 1982). In turn, we follow your advice to discuss the potential role of factors, such as need for cognition for how people do this task. In fact, this ties in nicely with recent work on the specificity with which people value (or don’t value) types of efforts. Recent work has shown that while people who are high in need for cognition prefer cognitively demanding tasks (and find them not boring), this does not translate to their valuation of physical effort. Likewise, valuing physical effort (measured with a scale that is similar to NfC scale) was linked to not finding sports boring and preferring physically effortful tasks. Interestingsly, network analyses and correlation analyses showed that valuing physical or cognitive effort are psychometrically disctinct latent constructs that only share a small to moderate correlation (Wolff, et al., 2023). We discuss this.**

“*To make sure that the participants' level of effort was not affected by rewards for good performance, they received a flat payment that was independent of their performance. Additionally, we provided no feedback on the quality of their performance in the physical task. Still, the feedback provided on mental task performance might have served as an incentive, enhancing the perceived rewards in the task configurations where a mental task is anticipated, leading to a higher effort investment. One factor that might contribute to higher force production in the trials where a mental task is anticipated* *might be “need for cognition” (Cacioppo et al., 1996; Mækelæ et al., 2023; Zerna et al., 2023). This refers to the tendency to engage in cognitively challenging tasks and university students tend to score high on this tendency to value cognitive effort (Cacioppo & Petty, 1982).Thus, one might speculate that the prospect of a mental task trial increased the reward value of the task, thereby licencing a relatively higher investment of effort. However, recent work that has investigated the domain-specificty of peoples’ effort perception showed that high need for cognition is not strongly related to peoples willingness to mobilize physical effort (Wolff, et al.,2023).* Taken together, these *results suggest that participants demonstrate a high flexibility in their effort regulation, making fine-graded adjustments based on the integration of various sensations. However, it is important to note that the magnitude of these effects is small,* and our results do not indicate that prospective mental effort factors are integrated into the computation of current physical effort costs in a straightforward way. Further research is needed to assess whether this is because people treat mental and physical effort as separate cost factors or due to methodological constraints in our research design. *In future studies, it will also be be interesting to assess how individual differences in peoples perception physical and mental effort alter the alignment between subjective effort and obkective performance across different effort domains*.”

**9. Figure 3: highlighting the information that is relevant for the reader would be helpful. Right now, it is hard to know where to look and where is the relevant information. An easy way could be to add dotted lines on the center and right panels to show where the initial force level (for the no-mental effort condition) lies for each RPE level, therefore facilitating the comparison. Currently, it’s almost impossible to compare the curves because of the size of the figure.**

**Thank you for your feedback regarding the clarity of Figure 3. We agree that it may be challenging for readers to grasp the relevant information. For that reason, we have decided to create a new figure to more clearly illustrate the differences in produced forces associated with the instructed physical effort levels, as well as the three-way interactions of force production, mental effort demand, and physical effort level. This was done by organizing according to physical effort levels rather than mental effort demands. The three-way interactions are more pronounced at higher levels of physical effort. The manner in which mental effort demands interact with the physical effort level varies over time, as evidenced by the differing slopes.**

**10. Force integration over 2s:**

**A) The authors report that the force used for calibration was just based on a peak force as is classically done in these type of experiments, but the force required during the task was based on an average over 2 seconds. What was the reason for this procedure and not using a peak force as well? It should be clearly acknowledged in the manuscript as it seems a bit awkward to me to not use the peak force as well in their analysis, which would also probably strongly reduce the delta between force produced and target force which is relatively gigantic right now (range of produced force is between 0 to 35%, while the required range was between 20 to 80%) because of the way produced force is computed.**

**We have carefully considered your remarks regarding the different computations of MVC, the produced submaximal forces, and the potential consequences for the results and our interpretations.**

**Our decision to use the averaging method is based on the assumption that participants need time to regulate their force outputs, especially when a paradigm such as the production method is used. Participants were asked to produce forces that reflected specific target RPE levels, and they were instructed to squeeze for a duration of 4 seconds. Also, regarding the task instructions, we do not assume that participants regulate their produced force in a way that they aim for the target level with a peak and then release when they have reached that peak. Unlike with MVC, the goal was not to exert maximal force but rather to sustain the force aligned with the RPE-target level for 4 seconds. Therefore, we assumed it is more of a gradual regulation process from below or above the target level rather than an aim for peak force. We averaged the force across 2 instead of 4 seconds because we assume that participants need time to react and stop in response to the target force instruction.**

**However, to ensure that the obtained results were not caused by the submaximal force computation method, we reanalyzed the data using peaks of the produced force. The delta between the produced force and the target level was slightly smaller, however not substantially (peak -> 20 %: M = 10.28% (SD = 6.03), 40%: M = 17.71% (SD = 8.06), 60%: M = 24.13 % (SD = 10.06), 80%: M = 38.28% (SD = 12.58%). For comparison also the averaged produced forces (%MVC): (20%: 8.81% (SD = 6.66), 40%: M = 15.07 (SD = 8.59), 60%: M = 20.69 (SD = 10.53), 80%: M = 33.14 (SD = 14.18)). The alignment revealed the same nonlinear pattern in the higher effort levels when the peak values as well as the averaged values, were used to compute the produced force (please see Figure)**

**Figure with peak produced force:**

**For this reason, we decided to stick with the average computation. However, we tried to make our reasons more clear. We added the detailed analyses with peak submaximal forces to the OSF.**

“Isometric hand grip MVC was defined as the peak force produced during three maximum contraction trials, whereas the measured force for the submaximal contractions, produced during the task, was averaged over two seconds. *These differences in calculation were made on the assumption that, using a production method, participants need time to regulate their force to produce a certain target RPE-level.* Therefore, due to these differences in force calculations, the produced force is not expected to reflect the equivalent percent relative to MVC. Simply put, if participants are asked to produce 60% of MVC, participants are likely to produce less than 60%.”

**11. Was there any calibration before the task to ensure that the participants understand what each level meant in terms of force production or are the authors relying on the fact that this should completely rely on their subjective feeling? Still, it seems strange that 80% of perceived exertion does not map above 35% of actual produced force. That would mean that any force produced above 35% of MVC would already feel extremely effortful (>80%) for the participants. It is possible, but it would be nice to 1) control that this is not due to the way force was extracted (not a peak but 2s average) and 2) that this is not due to the fact that participants are not incentivized to perform the task as final payment is fixed, so they could overrate how some force levels feel because they lack any incentive to produce force.**

**No calibration was conducted before the task. Participants were instructed to produce their MVC, which was then told to be an RPE of 10 (100%). They were informed that subsequent force production would be relative to this MVC as a subjective effort of 10. Therefore, participants relied solely on their subjective feeling during submaximal force production, aiming to exert a force equivalent to what they perceived as an 8 relative to their MVC feeling as a 10. We clarified that in the procedure section**

Following this baseline measure, the exact procedure was explained to the participants. *They were told to produce forces subjectively representing 20%, 40%, 60%, and 80% of their maximum, which should represent a subjective effort of 100% or a 10 on the RPE scale.* To facilitate understanding, participants were presented with the modified CR-10 scale, which displayed the respective percentages (20%, 40%, 60%, 80%) next to the target levels (2, 4, 6, 8). All individual target levels were accompanied by verbal descriptors (not strenuous, slightly strenuous, strenuous, very strenuous). They were instructed to squeeze for four seconds while the term "GO!" was displayed. For force procudtions participants should solely rely on their subjective feelings. *No feedback was provided on either the MVC measures or the submaximal measurements.*

**We agree that observing an average of 35% in produced force when participants were instructed to produce 80% of their MVC is unexpected. We controlled for differences in force calculation (averaging force over 2 seconds instead of peak) by redoing the analyses with peak force values instead of averages. The results differ insignificantly from the results obtained with the averaging method.**

- **10.28 % (6.10) in target level 20%.**
- **17.71 % (8.15) in target level 40%**
- **24.13 % (10.17) in target level 60%**
- **38.28 % (12.73) in target level 80%**

**Participants were made aware that they received flat payment for study participation, irrespective of their task performance. Therefore, we cannot rule out that the lack of incentive may led to an overestimation of certain subjective effort perceptions. However, research on similar tasks has also found that produced forces, are lower than those corresponding to the instructed subjective effort level (Cochrane et al., 2007; Pincivero et al., 2011, Myanmoto et al., 2020, Weilharter et al., 2024). As you suggest in your next comment, participants had no feedback on their physical task performance, giving them any possibility to adjust their produced force. This might explain why the actual produced force is substantially different from the required force.**

**12. Maybe I missed it, but it wasn’t clear to me if the participants get any feedback at all regarding the “true” force required to reach the corresponding levels during training. It seems to me it’s not the case, but it depends on whether the “practice trial” included feedback or not. If there was feedback, there is a high risk that people are closer to the feedback at the start of the experiment and just deviate from it with time-on-task. If not, then that would explain why the actual force is so far from the required force but it would be important to state it clearly in both cases.**

**No, participants did not get feedback regarding a “true” force per level. It is important to note that this was done because our research question focused on how one’s feeling (in terms of subjective effort) would translate to an objective outcome. In turn, providing an external anchor for any “true” force would be at odds with this research question (simply put, there is no true force that we can externally set, as the true force should reflect the true subjective experience of the person) and might even lead to the artifact that participants would adjuste their force according to this feedback. Indeed, recent work has shown that providing any feedback (e.g., a force line) alters the alignment between subjective effort and objective performance (Weilharter et al., 2024). To avoid this, we made sure that participants had no form of external feedback about their physical performance. Throughout the manuscript we have now taken great care to make this design aspect more explicit.**

**For example:**

“The experiment began after participants completed a practice *N-back trial, which aimed to familiarize themselves with the task. This trial could be repeated as often as necessary until the participant fully understood the task. There were no practice trials for the physical effort task.* The full experiment consisted of 6 blocks, 40 trials, each with a duration of 16 seconds. Blocks were separated by a resting period of 150 seconds to avoid excessive fatigue. The feedback provided was not tied to specific trials. Hence, participants were not given any information on their performance regarding different task difficulties. Importantly, in order to make participants truly rely on their internal sensations of effort, no feedback or monitoring of their physical performance was provided[…]

[…]They were instructed to squeeze for four seconds while the term "GO!" was displayed. *For force productions participants should solely rely on their subjective feelings. No feedback was provided on either the MVC measures or the submaximal measurements* […].”

**13.The mental effort conditions, both low and high, imply that subjects perform task switching regularly, a process known to be related to mental effort in itself, while the no-effort condition is just pure physical effort all along. Did the authors consider that in their analysis? Maybe a first analysis grouping low and high mental effort vs no-mental effort could be worth already to sit if there is a global effect of mental effort (task switching + N-back task) vs no mental effort. This is also important because the jump from no-mental effort to low-mental effort could be higher than from low-mental effort to high-mental effort, because of the task switching addition in the mental-effort condition and the possibility to rest (and therefore recover) in the no-mental effort condition. Plus the mental effort condition receives a feedback at the end of each block and not the other condition.**

**Thank you for bringing this to our attention. We agree that task-switching should be taken into consideration. While we did not observe a main effect of mental effort condition (no, low, high), indicating no difference in produced force between the three mental effort conditions when physical effort levels were averaged, we did calculate a Helmert Contrast to compare the force produced in tht trials with (easy, difficult) and without a mental task. Interestingly, this revealed a lower force in the condition of no mental effort. However, as there is no main effect of mental task, but a an interaction with physical task and temporal dynamics in this interaction, it seems that the obtained effects are not solely attributable to task-switching. and more complex factors might be integrated in the force investment. Aditionally, we did not observe such a force pattern in the three-way-interaction figure, which could be attributed to task-switching costs. Furthermore, we assume our experiemental paradigm to be quite nested, suggesting that task-switichinv cists might be present in all conditions, including the no mental effort condition.**

**Minor comments**

**1. The introduction, although very well written, clear and enjoyable to read, seemed a bit too long and off the topic of the article, with a long development on the expected value of control (EVC) which is not really implemented (at the computational level). I would center it a bit more on the main questions addressed in the manuscript with a clearer explanation of the premises of the authors (see previous comments).**

**Thank you for your positive feedback on the readability and comprehensiveness of our introduction. We agree that the section discussing the expected value of control might be too long, especially given that it is not computationally implemented in the present study. We have shortened this section and focused more on the main questions addressed in the paper.**

“Cognitive control, also referred to as self-control, is defined as "the set of mechanisms required to pursue a goal, especially when distraction and/or strong competing responses must be overcome" (Shenhav et al., 2013, p.217). Applying control is perceived as effortful (Cohen et al., 1990), and perceived effort is understood to index the costs of applying control (Shenhav et al., 2017). *In the present study, we adapt the definition of perceived effort as “the instantaneous experience of investing resources to perform an action” (Halperin & Emanuel, 2020, p.7). In line with this definition we understand the perception of effort to be based on the integration of various contributing factors rather than being solely contingent on objective performance (e.g., force production) (Halperin & Emanuel, 2020).*

*Motor control and cognitive control theories suggest that the execution of a control action reflects a reward-based choice that is geared towards minimizing control costs (Wolpert, 1997; Shenhav et al., 2013). To illustrate, according to one influential framework—the Expected Value of Control Theory (EVC theory)—the expected value of control is calculated for all available behavioral options by discounting the expected positive and negative payoffs that are associated with task engagement and the intrinsic cost of control. These intrinsic costs scale with task demands and task duration (Shenhav et al., 2013). Thus, harder and longer tasks are expected to be more costly than short and easy ones (Wolff et al., 2021). During task performance, environmental information (e.g., feedback, opportunity for rewards) and internal sensations (e.g., pain, motivational loss, boredom) are assumed to be monitored, updated, and integrated to initiate adjustments in effort allocation (Geana et al., 2016; Shenhav et al., 2013; Shenhav et al, 2016). Consequently, if changes in perceived control costs alter this cost-benefit analysis, performance decrements or even task termination are expected to ensue (Shenhav et al., 2013).*

Empirical evidence demonstrates that the regulation of mental and physical effort can be effectively described by theories relying on the concept of a cost-benefit analysis (Manohar et al., 2015; Wolff et al., 2020). However, recent theoretical and empirical advancements notwithstanding, the relationship between perceived effort as an index of control costs and the translation of this perception into objective behavioral output is not fully understood. Simply put, we do not know how perceived effort levels and objective output align and what factors affect this alignment. In this study, we will investigate these questions through the lens *of reward-based choices theories of control.*”

**2. The authors use the word “effort” to designate both the level of force produced (ie physical work or force produced) and the subjective feeling that accompanies it (effort). A clear distinction and definition of these two terms could be helpful to improve the clarity of the manuscript (see (Wolpe, Holton, Fletcher, 2024) and (Bermudez, Massin, 2022)).**

**We agree that this is very important, especially since participants produced actual physical work in our study, and it is only a means to understand if the subjective required effort level, i.e., perceived effort levels aligns with produced force in a linear fashion and whether this depends on anticipated further demands. To clarify this, we added an explicit definition of perceived effort (Halperin & Emanuel, 2020) and reviewed the manuscript to clarify whether we are referring to the actual produced force or the perceived effort.**

”*In the present study, we adapt the definition of perceived effort as “the instantaneous experience of investing resources to perform an action” (Halperin & Emanuel, 2020, p.7). In line with this definition we understand the perception of effort to be based on the integration of various contributing factors rather than being solely contingent on objective performance (e.g., force production) (Halperin & Emanuel, 2020).* ”

**3. Replacing the RPE levels 2/4/6/8 by the % of force (20/40/60/80%) in the manuscript and especially in the figures could help to improve the readability of the manuscript, especially since based on the authors the instructions were “how hard am I squeezing this handgrip dynamometer, relative to my maximum strength?” (Page 18) and that “To facilitate understanding, participants were presented with the modified CR-10 scale, which displayed the respective percentages (20%, 40%, 60%, 80%) next to the target levels (2, 4, 6, 8).” (Page 11).**

**Thank you. We have revised the figures and text accordingly*.***

**4. The authors decided to use “occurrence” to model the effect of time on the produced force. Did they also tried to use trial number instead? Since trials are interleaved, trial number might be more relevant as it should reflect cumulated fatigue. A nicer way to deal with fatigue though of course would have been to use a more complex model like in (Matthews et al, 2023) implementing recoverable (in the no-mental effort condition and between blocks) vs unrecoverable fatigue (accumulating over all trials) but a first test with trial number would already be interesting.**

**Thank you for bringing this more complex approach to our attention. Occurrence, or in our terms, the Nth event of task combination, refers to the number of times a specific task combination occurs. For example, physical effort level 80% with no mental effort task. Each mental and physical task combination occurred 20 times but in a randomized order. If we were to examine the produced force across trials, we agree that fatigue could perhaps be depicted more clearly. However, for our research question (i.e., does produced for ce change within a condition), we feel Nth event seems to be a suitable analysis. Both, with respect to answering our research question and ease of interpretation/communication to readers.**

**5. Page 4: “During task performance, the EVC is constantly monitored, updated, and adjusted if needed.” Based on what are the authors making that statement? As far as I know, there is not much evidence proving that for now so I would not make strong statements about the EVC computation being constantly monitored, especially since this does not appear to add anything relevant for the current study.**

**Thank you. We agree and have removed the sentence.**

**6. Page 8: Inclusion/exclusion criteria are missing. The authors just state that participants had to speak German and to not have any arm injuries. What was the age range for the study? Were there any other criteria (related to pathologies for example)?**

**Thank you for pointing out that some important information regarding the sample is unclear. There was no predetermined age range for participation. Participants voluntarily registered for the study. The data was collected at the University of Konstanz, and all participants were adult university students (Min = 18, Max = 32 years). Apart from speaking German and having no arm injuries, no exclusion criteria were used. We also did not screen for pathologies. We added these information to the methods section.**

“A total of *N* = 44 *adult university students (f* =18, *m* = 26) took part in the study (*M* = 23.96 ± 2.66 years old, *Min = 18, Max = 32). Participants were recruited to participate in a study, where they receive a flat payment of 20€ for their participation.* Only participants who spoke German as their first language and had no preexisting hand or arm injuries were included. *No further inclusion or exclusion criteria were used.”*

**7. Page 8: The data was apparently acquired between 11/20 and 07/22, was there any covid-related information that would be important to reveal? Like were the participants wearing a mask during the experiment (possibly impacting respiration and effort production) or not? And if so, was it the case for all of them or only a subpart? This is not essential but it would be nice to acknowledge.**

**Thank you for drawing our attention to some possible COVID-related issues. Data was collected from 12/21 to 04/22. Due to the restrictions at that time, all participants had to wear a mask during the experiment. Since everyone wore a mask, we do not assume that potential impacts on respiration and effort production would confound the results. Because of this, we decided not to mention it in the actual manuscript. However, if you consider it important, we would be happy to include it in a future revision.**

**8. Page 9: when the measure of MVC is introduced, this sentence “whereas the measured force for the submaximal contractions was averaged over two seconds” should make it clear that it’s talking about the measured force “in the task”.**

**Thank you, we added this information.**

“Isometric hand grip MVC was defined as the peak force produced during three maximum contraction trials, whereas the measured force for the submaximal contractions, *produced during the task* was averaged over two seconds.”

**9. Page 12: “After data acquisition, handgrip measurements and experimental instructions were merged and down-sampled to ten rows per second.” This sentence is unclear. It should probably state “handgrip measurements and experimental instructions periods” but it would probably be easier to explain if the authors give a name to each timeperiod in Figure 1 and then refer to it here.**

**Thank you. We agree that this sentence is unclear. It refers to a technical issue where PowerLab sampled the data at a high frequency, resulting in hundreds of measurements per second. We decided to downsample the data to 10 measurements per second. Since this information refers to data preprocessing and specific measurement settings and does not add value or have relevance for the reader in understanding the statistical analyses, we decided to remove this sentence to reduce potential confusion.**

**10. Page 12: “The experiment began after completing a practice trial, which could be repeated as often as necessary.” What was this practice trial consisting on exactly? Just squeezing the handgrip? Did they get any feedback or not? Was there any reason for the experimenter to ask the subjects to repeat this practice trial or is solely based on the participants’ willingness to explore how the grip works?**

**Thank you for these important questions. The practice trial only consisted of an N-back trial. Participants did not receive any feedback for the practice trial. There was no practice trial for the handgrip task. We added this information in the “Procedure” section.**

“The experiment began after participants completed *a practice N-back* trial, *which aimed to familiarize themselves with the task.* This trial could be repeated as often as necessary *until the participant fully understood the task. There were no practice trials for the physical effort task.* “

**11. Page 13: how was mental effort encoded? Was it as 0/1/2 levels corresponding to no/low/high mental effort? The description is clear for the physical but not for the mental.**

**The mental effort levels were initially coded as 1 (no), 2 (low), and 3 (high). After the preprocessing steps, the encoding was changed to no, low, and high. Participants were not aware of this encoding or the differentiation of no, low, and high mental effort. They were only told that if a mental task was to be performed, it would be either easy or difficult. The encoding for the physical effort task corresponds to the RPE target levels.**

**12. Page 14: “Interestingly, no significant differences were found between mental (M = 4.10, SD = 1.98) and physical (M = 4.47, SD = 2.08) exhaustion ratings, indicating that the procedure caused a moderate overall fatigue level.” I understood that fatigue ratings were asked regularly before and after each of the six blocks so it would be nice to clarify what this measure is referring to. Is it just the final fatigue rating? What would be more meaningful would be the delta between the final and the initial fatigue rating for each dimension (ie compare [final fatigue rating-initial fatigue rating] for mental vs physical) which is not the final state but how much the task increased the fatigue ratings.**

**Thank you for bringing this to our attention. Participants were asked to rate their physical and mental fatigue after each experimental block, i.e., after 40 trials. The provided mental and physical fatigue ratings are the averages across all 6 blocks. On a 10-point rating scale, participants reported an average fatigue of 4.10 (1.98) in the mental and 4.47 (2.08) in the physical domain, indicating an overall moderate level of fatigue.**

**Although the overall fatigue was moderate, we agree that examining the change in experienced fatigue from the initial to the final rating would enhance our understanding of fatigue as a contributing factor to force decrements over task duration. Indeed, the paired sample t-test showed a significant increase in experienced fatigue in both domains from after the first block to the end of the experiment, i.e., after all 6 blocks had been performed. Unfortunately, we did not measure a baseline fatigue level prior to the first block.**

**Given that the experiment involved a physically and mentally fatiguing task, lasted approximately two hours, and included a substantial number of trials, we did anticipate a significant increase in fatigue. However, in both domains, the experienced fatigue did not exceed 5.3. We added this information.**

“*Participants reported overall increasing fatigue over the task duration. Mental fatigue increased significantly from the first block (M = 2.79, SD = 1.71) to the sixth block (M = 4.98, SD = 1.99) (t(42) = 9.18, p < .001). Similarly, physical fatigue ratings showed a significant increase from after the first block (M = 3.53, SD = 2.00) to the end of the experiment (M = 5.19, SD = 2.26) (t(42) = 5.26, p < .001). However,* across all blocks, participants reported a moderate fatigue level in the mental (*M* = 4.10, *SD* = 1.98) and physical (*M* = 4.47, *SD* = 2.08) domains, indicating that the procedure caused a moderate overall fatigue level. *This indicates that* participants were unlikely to be too exhausted to produce the required forces throughout the experiment. *In turn,* potential declines in produced force are *un*likely *to be purely due to accumulated physical fatigue, but might also reflect changes in participants cost-benefit analyses for exerting effort towards the task.* “

**13. Page 22: “Current research argues that force accuracy between intended and actual force can be increased by visual feedback (Kim et al., 2021).” The authors could also expand a bit on the fact that individuals vary widely in their metacognitive and interoceptive capabilities. Therefore, strong interindividual differences could also be present across the participants studied based on this.**

**Thank you for bringing to our attention that we could discuss differences in interoceptive and metacognitive capabilities regarding the question of alignment. In consideration of this, we decided to add the individual data points of each participant in Figure 2. By doing so, we observed that most participants showed a similar pattern regarding the alignment, even though they might differ in their interoceptive abilities. However, we agree that in future studies, one should consider and perhaps add a measurement so this matter can be discussed accordingly.**

“Further research is needed to assess whether this is because people treat mental and physical effort as separate cost factors or due to methodological constraints in our research design. *In future studies, it will also be be interesting to assess how individual differences in peoples perception physical and mental effort alter the alignment between subjective effort and objective performance across different effort domains.*“

**14. It is known that males generally have a higher strength and MVC than females. It would be interesting to verify whether the main results reported in the study are also valid when one splits the data between male and female just to be sure that differences in capacity and/or in sex do not influence the results presented, but this could go in the supplementary material.**

**Thank you. We agree that it would be interesting to control sex to see whether the main results are supported. However, in the present study, we focus on a within-subject protocol and the alignment of produced force relative to individual capacity. Therefore, we are concentrating on relative produced forces rather than absolute values, as the %MVC is standardized for each participant’s individual capacity.**

**15. Was there any measure to prevent participants from doing a force below their actual MVC during calibration or, at least, to debrief at the end of the experiment if they understood that this procedure was to calibrate their MVC on which the rest of the task would be based? In these tasks, sometimes participants understand that this is a calibration and they perform below their actual MVC to make the task easier for them. The answer to my previous comment regarding the instructions for the final reward would also help to answer here as, if they know that the final payment is fixed, they also have no reason to cheat their MVC calibration, but this is an important detail.**

**There was no measure to prevent participants from exerting a force below their actual MVC. However, we do not assume that participants deliberately chose to squeeze less than their actual MVC. Prior to the MVC measurement, they were not aware that this was intended to be a calibration method. They were simply instructed to “squeeze as hard as they can” without any information about the subsequent steps of the experiment. After the initial MVC measurements, they were informed that the subjective effort they experienced during this MVC should correspond to a 10 on an RPE scale and that subsequent forces should be produced relative to this. Additionally, participants knew that the reward was based on study participation and not on performance, giving them no reason to intentionally produce a lower force than their MVC. We clarified that in the procedure section.**

“Following this baseline measure, the exact procedure was explained to the participants. *They were told to produce forces subjectively representing 20%, 40%, 60%, and 80% of their maximum, representing a subjective effort of 100% or a 10 on the RPE scale.* To facilitate understanding, participants were presented with the modified CR-10 scale, which displayed the respective percentages (20%, 40%, 60%, 80%) next to the target levels (2, 4, 6, 8). […]”

“[…] After the data collection, participants were debriefed*, and* *regardless of their task performance,* they received 20€ for their participation. The whole experiment lasted around 120 minutes. “

**Thank you for thoroughly reviewing our manuscript and providing such detailed feecback. We greatly value your insights regarding additional literature and your promising ideas to improve our data analysis and theoretical reasoning. We have taken your feedback into careful consideration and hope we have adequately addressed your comments.**
